# A Simple Method of Reducing Coolant Leakage for Direct Metal Printed Injection Mold with Conformal Cooling Channels Using General Process Parameters and Heat Treatment

**DOI:** 10.3390/ma14237258

**Published:** 2021-11-27

**Authors:** Chil-Chyuan Kuo, Shao-Xuan Qiu

**Affiliations:** 1 Department of Mechanical Engineering, Ming Chi University of Technology, New Taipei City 243, Taiwan; M05118021@mail.mcut.edu.tw; 2 Research Center for Intelligent Medical Devices, Ming Chi University of Technology, New Taipei City 243, Taiwan

**Keywords:** direct metal printing, maraging steel powder, coolant leakage, heat treatment, precipitate

## Abstract

Direct metal printing is a promising technique for manufacturing injection molds with complex conformal cooling channels from maraging steel powder, which is widely applied in automotive or aerospace industries. However, two major disadvantages of direct metal printing are the narrow process window and length of time consumed. The fabrication of high-density injection molds is frequently applied to prevent coolant leakage during the cooling stage. In this study, we propose a simple method of reducing coolant leakage for a direct-metal-printed injection mold with conformal cooling channels by combining injection mold fabrication with general process parameters, as well as solution and aging treatment (SAT). This study comprehensively investigates the microstructural evolution of the injection mold after SAT using field-emission scanning electron microscopy and energy-dispersive X-ray spectroscopy. We found that the surface hardness of the injection mold was enhanced from HV 189 to HV 546 as the Ni-Mo precipitates increased from 12.8 to 18.5%. The size of the pores was reduced significantly due to iron oxide precipitates because the relative density of the injection mold increased from 99.18 to 99.72%. The total production time of the wax injection mold without coolant leakage during the cooling stage was only 62% that of the production time of the wax injection mold fabricated with high-density process parameters. A significant savings of up to 46% of the production cost of the injection mold was obtained.

## 1. Introduction

Additive manufacturing (AM) [1,2] technology has been widely used to produce prototypes and physical models in industry because it has the capacity to manufacture components with sophisticated geometries. Metal AM technology in particular has received much attention, in techniques such as selective laser melting [3], direct metal laser sintering, vacuum diffusion bonding, selective laser sintering, and selective electron beam melting. Additionally, it can be used to manufacture injection molds [4] for the mass production of new products. Mazzarisi et al. [5] studied three main process parameters of direct laser metal deposition and suggested the best ranges of process parameters to establish the most suitable parameters for predicting the geometric characteristics of clad. The results showed that new formulations were in good agreement with the behaviors defined in the literature. Contaldi et al. [6] investigated the effects of powder reuse for two kinds of precipitation-hardening stainless steel. They found that reusing the excess metal powder in powder bed fusion processes is possible and no significant variation was observed for the martensitic phase. Alafaghani et al. [7] studied the effects of manufacturing procedures on the mechanical properties of metal laser sintering parts. The results showed that IN718 and 15-5PH can be used in applications with elevated environmental temperatures. AlMangour et al. [8] investigated the deformation phenomena of 17-4 precipitate-hardening stainless steel manufactured by direct metal laser sintering using transmission electron microscopy and micropillar compression. The results showed that the microstructures and properties of 17-4 stainless steel specimens fabricated by direct metal laser sintering varied significantly from those of specimens produced by conventional methods. Kundu et al. [9] manufactured titanium nitride-reinforced alloy-based metal matrix composites using a fiber laser. The results showed that the microhardness was improved with increasing volume percentage of TiN.

The conformal cooling channel (CCC) is frequently employed in injection molding because of its more uniform cooling in comparison to conventional straight cooling channels during the cooling stage. An injection mold with the appropriate CCC is considered to be a piece of technology that can reduce cycle time. The manufacturing of an injection mold with CCC is affordable due to the recent developments in AM that provide increased design freedom, less wasted material, and minimized machining. In practice, coolant leakage will appear when the injection mold is not fabricated with high-density process parameters. The cooling time is affected by coolant leakage. This results in a defect named short shot: when the molded wax pattern is observed. To solve this major disadvantage, optimum process parameters are widely applied to manufacture high-density injection molds. A distinct disadvantage is that the process window is very narrow, which leads to a high failure rate in mold making when using direct metal printing technology; furthermore, it is a time-consuming process. Thus, a simple method of reducing coolant leakage is proposed in this study, integrating injection mold fabrication with general process parameters as well as solution and aging treatment (SAT). We evaluate the surface hardnesses of the test specimens before and after heat treatment [10,11]. A coolant leakage test experiment is performed to evaluate the effectiveness of the fabricated injection molds before and after heat treatment. To propose a mechanism for reducing coolant leakage after SAT, we perform a comprehensive investigation of the microstructural evolution of the injection mold after heat treatment using field-emission scanning electron microscopy (FE-SEM). Finally, the mechanism of the reduced coolant leakage is proposed on the basis of the microstructure evolution after heat treatment.

## 2. Experimental Details

**Figure 1:**
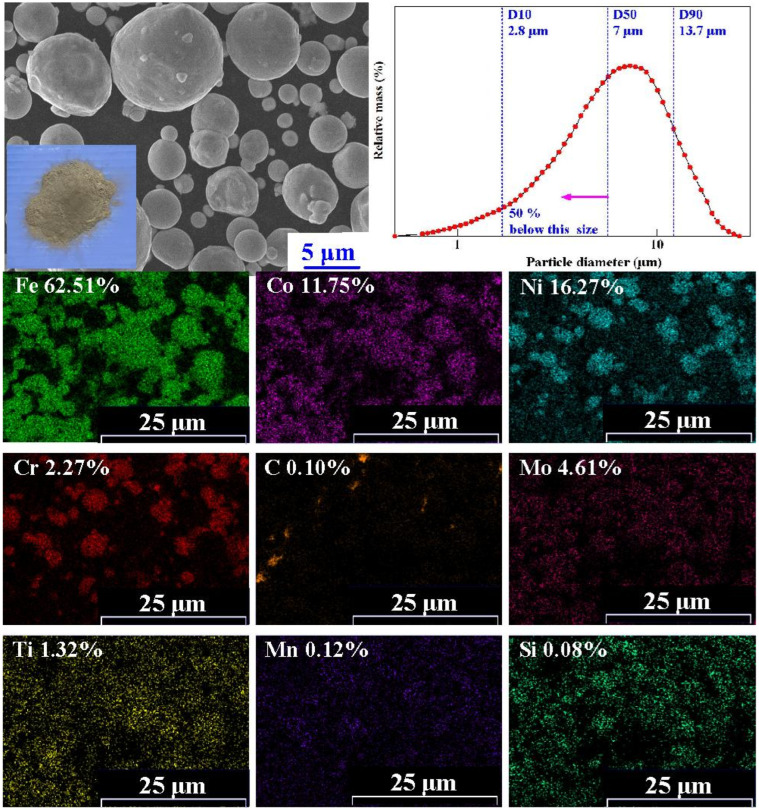
FE-SEM and EDS images of maraging stainless steel powder.

The geometric models of the injection-molded part, CCC, and injection mold proposed in this work were designed using computer-aided design software (Cero, parametric technology corporation Hsinchu, Taiwan). Selective laser melting (SLM) is a metal AM technology that uses a powder bed with a source of heat to create a metal mold or die. Stainless-steel powder (LaserForm^®^ 18Ni-300 Inc. South Carolina, USA) was used to manufacture injection molds using an SLM machine with a building volume of 100 × 100 × 80 mm^3^ (ProX 100, 3D System Inc. c. South Carolina, USA), equipped with an optical-path transmission system, a f-θ lens, a scanning galvanometer mirror, and a Q-switched ytterbium-doped yttrium aluminum garnet 50 W fiber laser with a wavelength of 1070 nm. Figure 1 shows FE-SEM (JEC3000-FC, JEOL Inc., Tokyo, Japan) and X-ray spectroscopy (EDS) (D8 ADVANCE, Bruker Inc. Massachusetts, USA) images of maraging stainless steel powder. Table 1 summarizes the chemical composition of the maraging stainless steel powder. The chemical composition of the maraging stainless steel powder included 11.75% Co, 62.51% Fe, 16.27% Ni, and 4.61% Mo, characterized using an EDS and FE-SEM. Three values, that is, D10, D50, and D90, are widely applied to characterize the size distribution of maraging stainless steel powder. D10, D50, and D90 are approximately 2.8 µm, 7 µm, and 13.7 µm, respectively. In general, D10 is defined as the point on the distribution curve where 10% of the particles fall. Both D50 and D90 are thus defined as points along the distribution curve that fall below 50% and 90%.

**Figure 2:**
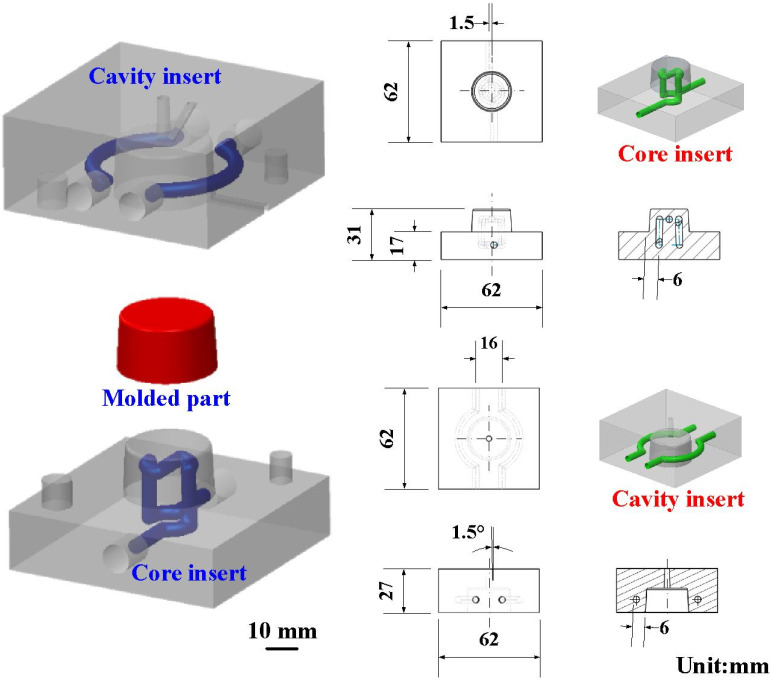
Details of injection mold with CCC.

**Figure 3:**
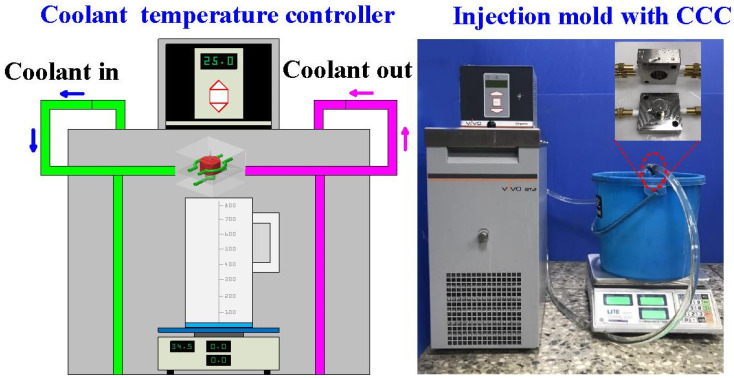
Experimental setup for investigating the amount of coolant leakage using a precision electronic scale.

**Figure 4:**
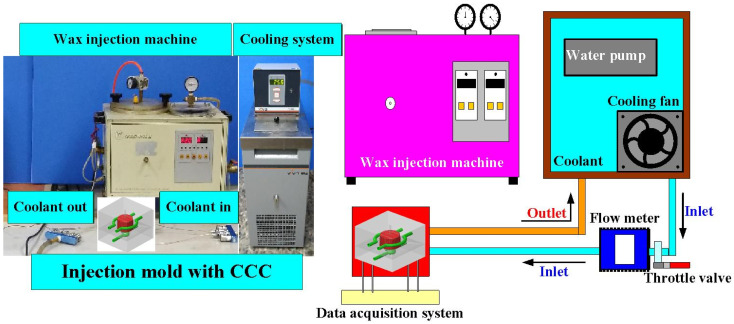
Experimental setup for evaluating the effectiveness of the injection mold before and after heat treatment.

To evaluate the Vickers hardness of test specimens after heat treatment, a cylindrical test specimen was designed with a height of 20 mm and a diameter of 20 mm. Figure 2 shows details of the injection mold with CCC. The injection-molded part is a pipe cap, which could be applied as a wax pattern for investment casting. The height, outer diameter, and thickness of the wax pattern were 15 mm, 23 mm, and 1 mm, respectively. The center distance with respect to the mold cavity was 6 mm and the diameter of the CCC was 4 mm. A circular CCC was used in this study because it has the smallest pressure loss during the cooling stage. The length, width, and height of the cavity insert were 62 mm, 62 mm, and 27 mm, respectively. The length, width, and height of the core insert were 62 mm, 62 mm, and 31 mm, respectively. Table 2 shows the process parameters of 3D printing. According to the results from a series of experiments, the process parameters for fabricating the high-density injection mold involved a layer thickness of 30 µm, laser power of 50 W, hatching space of 60 µm, and scanning speed of 200 mm/s. The injection mold with CCC was manufactured by general process parameters (i.e., a layer thickness of 50 µm, laser power of 40 W, hatching space of 100 µm, and scanning speed of 240 mm/s). To propose a simple method for reducing coolant leakage by integrating injection-mold fabrication with general process parameters and heat treatment, a series of experiments through three different heat treatment methods were performed. The experiments included solution treatment (ST), direct aging treatment (AT), and solution and aging treatment (SAT). Based on a review of the literature, various process parameters can be used for heat treatment. Some examples are given in the following. The ST temperatures can be selected as 1020 °C, 960 °C, 900 °C, 840 °C, or 780 °C and the durations can be 4, 2, 1, 0.5, or 0.25 h. The AT temperature can be selected as 560 °C, 520 °C, 480 °C, or 440 °C, and the duration can be 12, 9, 6, 3, or 1 h. General AT heat treatment procedures involve 900 °C followed by 400 °C, 900 °C followed by 440 °C, 900 °C followed by 480 °C, 900 °C followed by 520 °C, or 900 °C followed by 560 °C for 6 h [12]. In another study, the ST heat treatment was selected as 820 °C for 1 h and the AT treatment was selected as 460 °C for 5 h [13]. The AT temperature of 490 °C was applied in [14]. In a separate study, the AT temperature of 840 °C was applied, followed by the temperature of 480 °C [15]. As another example, an AT heat treatment was 510 °C for 1 h in [16]. In this study, the water was selected as the coolant. Figure 3 shows the experimental setup for investigating the amount of coolant leakage using a precision electronic scale. Figure 4 shows the experimental setup for evaluating the effectiveness of the injection mold before and after heat treatment. To evaluate the effectiveness of the injection mold before and after heat treatment, low-pressure injection molding was performed. Table 3 shows the boundary conditions used in low-pressure wax injection molding. The parameters included an injection pressure of 0.06 MPa, a fill time of 2 s, a coolant temperature of 25 °C, a coolant flow rate of 4 L/min, a mold temperature of 27 °C, an injection temperature of 98 °C, and a cycle time of 49 s. Wax (K512, Kato Taoyuan city, Taiwan) was used as a molding material to fabricate wax patterns via a low-pressure wax injection molding machine (0660, W&W Taoyuan city, Taiwan). A homemade cooling system was implemented, which was composed of a temperature controller (JCM-33A, Shinko New Taipei, Taiwan) and a thermo-electric cooler (TEC12706AJ, Caijia New Taipei city, Taiwan), three k-type thermocouples (C071009-079, Cheng Tay New Taipei city, Taiwan), as well as a data acquisition system (MRD-8002L, IDEA System New Taipei, Taiwan). To investigate the surface hardness of the test specimens after heat treatment, a Vickers hardness tester was used with an applied force of 9.81 N. To investigate the mechanism of the reduced coolant leakage of an injection mold after SAT, a comprehensive investigation of the microstructural evolution was performed using XRD and FE-SEM.

## 3. Results and Discussion

**Figure 5:**
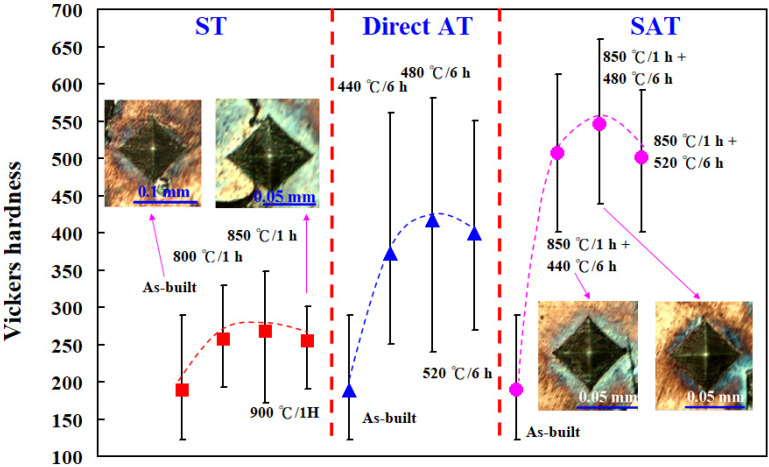
Vickers hardness of the test specimens after heat treatment.

**Figure 6:**
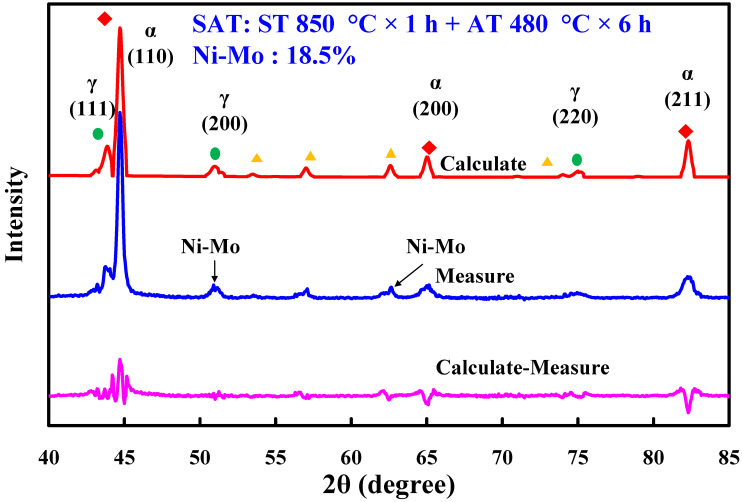
X-ray diffraction patterns of the test specimen processed by SAT with the optimal procedures.

Figure 5 shows the Vickers hardness of the test specimens after heat treatment. Three phenomena were observed. First, the optimum heat treatment procedure was ST at 850 °C for 1 h, followed by AT 480 °C for 6 h. Second, the surface hardness of test specimens after SAT was highest, followed by DAT. Third, the surface hardness of test specimens was highest after ST at 760 °C for 1 h. The most distinctive feature in this figure is that the highest test specimen Vickers hardness of approximately HV 546 was obtained via SAT with the optimum HT procedure. The Vickers hardness of the as-built injection mold was approximately HV 189. It can be clearly seen that the surface hardness of the injection mold was enhanced from HV 189 to HV 546. The increment in the surface hardness of the injection mold was about 198%. Figure 6 shows the X-ray diffraction patterns of the test specimen processed by SAT with the optimized procedure. The surface hardness was enhanced by precipitation of 18.5% Ni-Mo alloy after SAT heat treatment [17]. Note that the surface hardness obtained can meet the requirement of injection molds for plastic injection molding.

**Figure 7:**
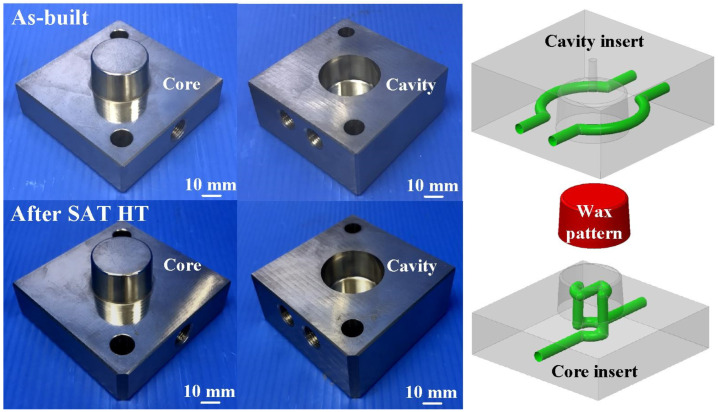
Two pairs of injection molds before and after SAT heat treatment.

**Figure 8:**
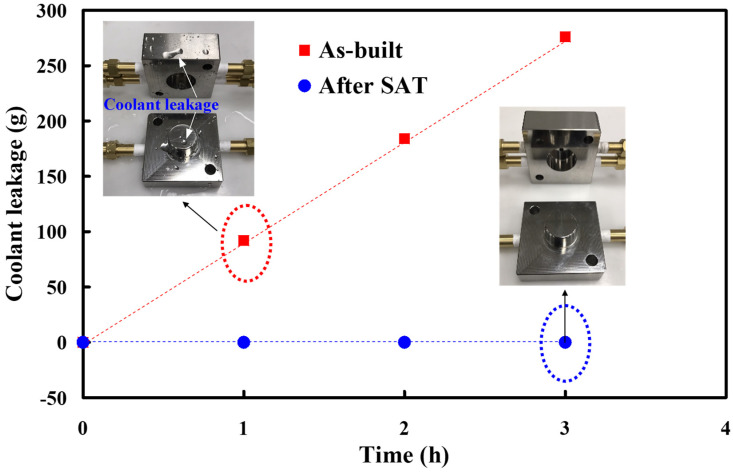
Results of coolant leakage test for an injection mold with CCC.

Figure 7 shows two pairs of injection molds before and after SAT heat treatment. After the optimum heat treatment procedure, post-process finishing operations used a precision milling machine to obtain the desired dimensions of the injection mold. Figure 8 shows the results of the coolant leakage test for an injection mold with CCC. As expected, there was no coolant leakage for the injection mold with CCC after SAT at 480 °C for 6 h. The proposed method solved the coolant leakage problem with no detrimental effects on the functions of either the injection mold or the CCC. However, the as-built injection mold had coolant leakage of approximately 276 g, 184 g, and 92 g after a test time of 3, 2, and 1 h, respectively. This indicates that the coolant leakage for a direct-metal-printed injection mold with CCC can be reduced by combining injection-mold fabrication with general process parameters and SAT.

**Figure 9:**
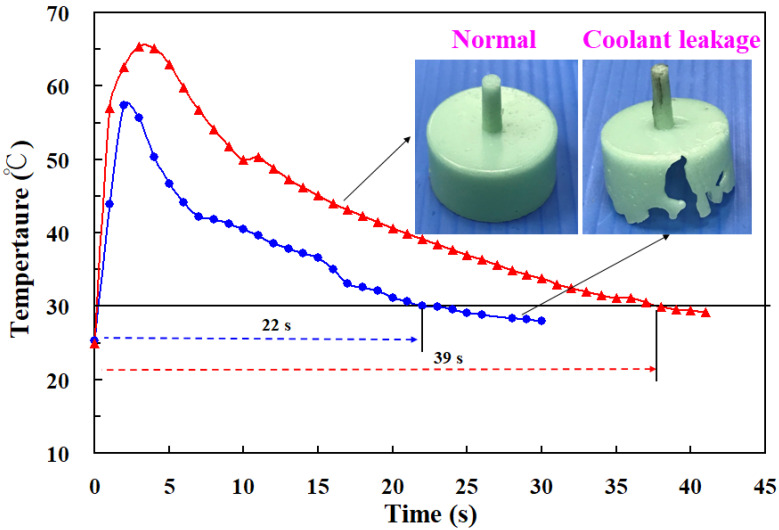
Cooling time of wax patterns for injection molds.

**Figure 10:**
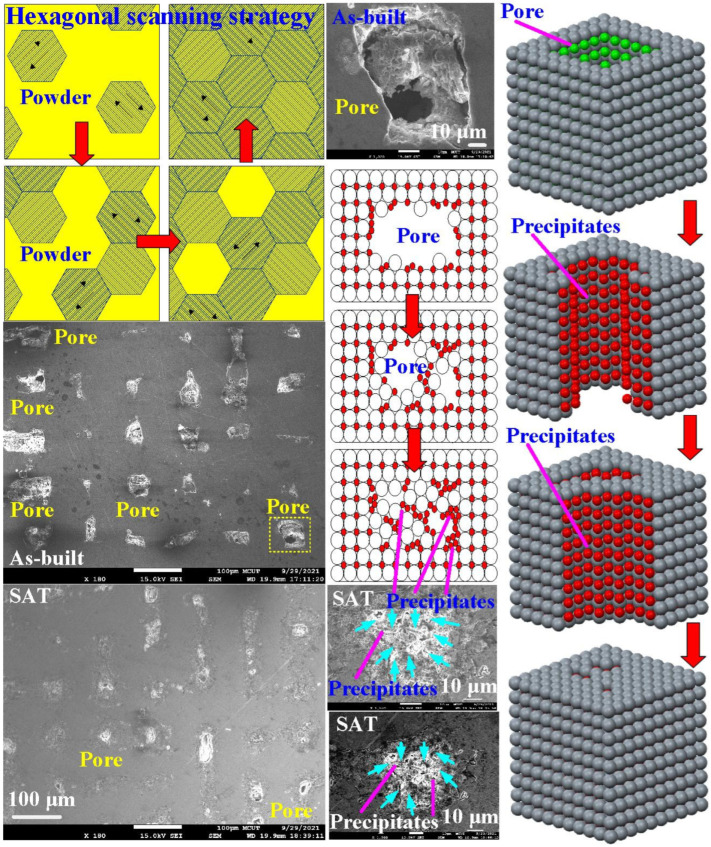
Microstructural evolution of the injection mold after SAT with the optimized procedure.

**Figure 11:**
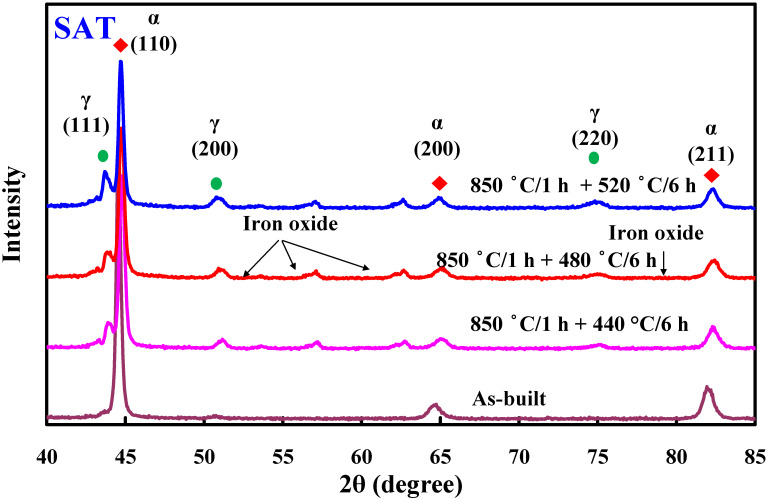

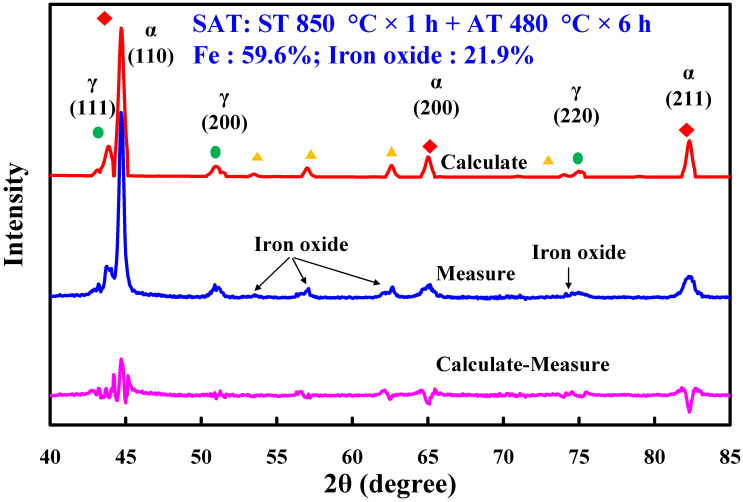
Occurrence of iron oxide precipitates after heat treatment.

**Figure 12:**
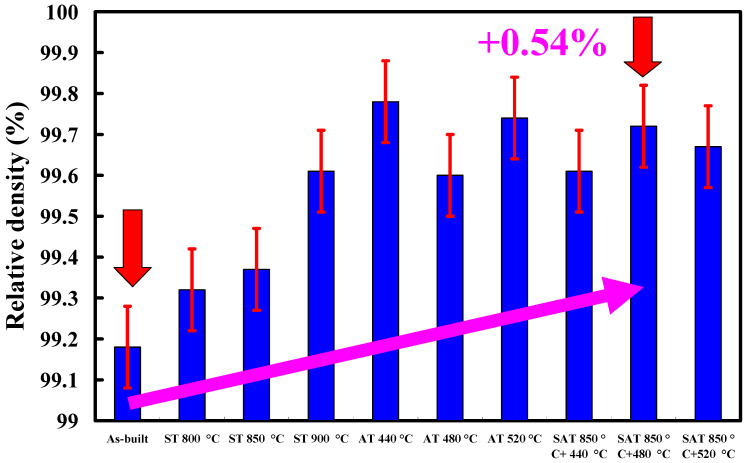
Relative densities of injection molds.

Wax patterns can be fabricated through low-pressure wax injection molding. We carried out wax injection molding to study the difference in the cooling times of the wax pattern for injection molds with and without coolant leakage. Figure 9 shows the cooling times of the wax patterns for injection molds. The cooling times of the wax patterns fabricated by the injection mold with and without coolant leakage were 22 s and 39 s, respectively. Note that a distinct defect of the molded wax pattern, named short shot (an incompletely filled mold cavity [18,19]), was observed when the mold had coolant leakage. In this study, injection molds were fabricated by a hexagonal scanning strategy because it provides a regular arrangement of pores inside the injection mold. Figure 10 shows the microstructural evolution of the injection mold after SAT with the optimized procedure. The most distinctive feature in this figure is that sizes of the pores were reduced significantly after SAT with optimal process parameters because iron oxide was precipitated, as shown in Figure 11. Note that the location of iron oxide precipitate was in the vicinity of the pores. This led to the absence of injection mold coolant leakage in the cooling stage after wax injection molding. Figure 12 shows the relative densities of the injection mold. The relative density of the as-built injection mold was approximately 99.18%. It can be concluded that the relative density of the injection mold processed by SAT with the optimal procedure was approximately 99.72%. The relative density of the injection mold was increased from 99.18 to 99.72%. It is interesting to note that the increment in relative density of the injection mold was about 0.54%.

**Figure 13:**
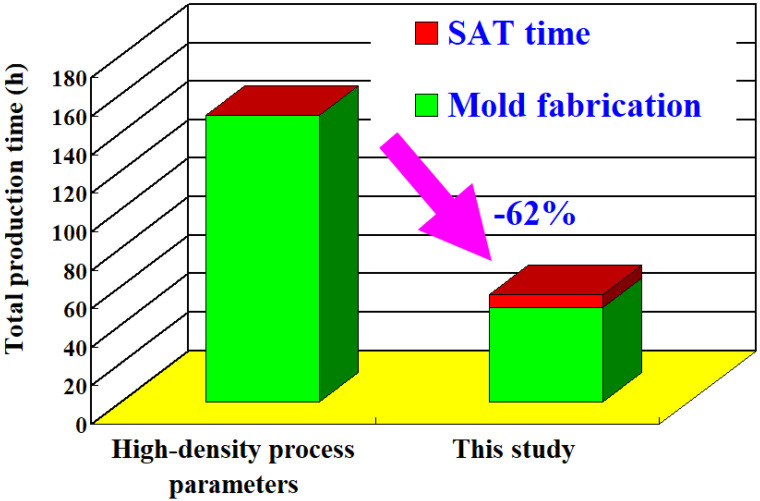
Total production time of an injection mold fabricated by high-density process parameters and the process parameters utilized in this study.

Figure 13 shows the total production time of injection molds fabricated by high-density process parameters and the process parameters utilized in this study. The production time of a high-density injection mold is about 149 h, taking 65 h and 84 h to produce the core and cavity mold inserts, respectively. Note that the fabrication time of an injection mold using the process parameters utilized in this study was about 49 h, taking 23 h and 26 h to produce the core and cavity mold inserts, respectively. The heat treatment time using SAT with the optimized procedure is about 7 h. Accordingly, the total production time to manufacture an injection mold without coolant leakage is only 56 h. This indicates that by using the method proposed in this study, a significant reduction in the total production time of approximately 62% can be obtained. Furthermore, the total production cost per high-density injection mold is about NTD 63,063 (New Taiwan dollars). However, using the process parameters utilized in this study, the total production cost of an injection mold is only approximately NTD 33,063. This means a significant savings of up to 46% in the total production cost of an injection mold can be obtained.

According to the results described above, the remarkable findings of this study have great practical value and show excellent potential for application in the precision investment casting industry [20,21]. The main contribution of this study is the proposal of a low-cost and highly efficient method to prevent coolant leakage during wax injection molding and 3D-printed conformally cooled injection molds. In this study, stainless-steel powder is applied to manufacture an injection mold. Different kinds of steel powders, such as Inconel 625, Ti_6_Al_4_V, Al-Si alloy, Ni-Ti alloy, 304 stainless steel, 17-4 PH stainless steel, 316-L stainless steel, CoCrMo, or Al-Fe-V-Si can also be applied to manufacture functional components, molds, or dies for industrial applications. These tasks are currently being investigated and the results will be presented in further studies.

## 4. Conclusions

Direct metal printing is a useful three-dimensional printing technology that can build any metal component with sophisticated geometry by melting metal powder layer by layer. In practice, two disadvantages of fabricating a high-density injection mold to prevent coolant leakage during the cooling stage are the narrow process window and the length of time consumed. The main findings and contributions of this study are summarized as follows:

1. This study shows great potential for application in the mold industry for its improved fabrication of an injection mold in terms of economics, efficiency, and speed. This simple method is applicable to any direct-metal-printed injection molds that incorporate CCC.
2. We comprehensively investigated and described the mechanism of the prevention of coolant leakage in direct-metal-printed injection molds with CCC.
3. The proposed simple approach solves the coolant leakage problem without any detrimental effects on the functions of either the CCC or the injection mold. The surface hardnesses of the injection mold were improved from HV 189 to HV 546 due to the Ni-Mo precipitates, increasing from 12.8 to 18.5%. The pore sizes were greatly reduced due to the iron oxide precipitates and the surface hardness of the injection mold was increased from 99.18 to 99.72%.
4. The total production time of a wax injection mold without coolant leakage was only 62% that of a wax injection mold fabricated with high-density process parameters. In addition, up to 46% of production cost savings could be obtained when an injection mold is fabricated using general process parameters.

## Figures and Tables

**Table 1:** Chemical composition of the maraging stainless steel powder.

Element	Ni	Co	Mo	Ti	Cr	Mn	Si
Wt.%	16.27	11.75	4.61	1.32	2.27	0.12	0.08

**Table 2:** Process parameters of 3D printing.

	High Density	This Study
Hatching space (µm)	60	100
Layer thickness (µm)	30	50
Laser power (W)	50	40
Scanning speed (mm/s)	200	240

**Table 3:** Boundary conditions used in low-pressure wax injection molding.

Parameter	Value
Injection pressure (MPa)	0.06
Fill time (s)	2
Coolant temperature (°C)	25
Coolant flow rate (L/min)	4
Mold temperature (°C)	27
Injection temperature (°C)	98
Cycle time (s)	49

## Data Availability

Data sharing is not applicable.
